# Morphological variation in the *Rhododendron pseudochrysanthum* Hayata (Ericales, Ericaceae) species complex from Taiwan

**DOI:** 10.3897/phytokeys.271.175682

**Published:** 2026-03-06

**Authors:** Ya-Rong Zheng, Yen-Hsueh Tseng, Hsy-Yu Tzeng

**Affiliations:** 1 Department of Forestry, National Chung-Hsing University, No. 145, Hsing-Ta Rd., Taichung 402, Taiwan Taiwan Forestry Research Institute Taipei City Taiwan; 2 Taiwan Forestry Research Institute, No. 53, Nanhai Rd., Zhongzheng Dist., Taipei City, 10066, Taiwan Department of Forestry, National Chung-Hsing University Taichung Taiwan

**Keywords:** Flower buds, pollen, seeds, statistical analysis, taxonomy

## Abstract

Our study examines the morphological and statistical differentiation within the *Rhododendron pseudochrysanthum* species complex through comparative analyses of macro- and micro-morphological characters. Using significance testing and cluster analysis, our results demonstrate that *R. pseudochrysanthum* Hayata ssp. morii (Hayata) Yamazaki var. *taitunense* Yamazaki is distinct from other members of the complex, namely *R. morii* Hayata, *R. pseudochrysanthum* Hayata, and *R. hyperythrum* Hayata. This taxon is characterized by glabrous mature leaves with revolute margins, larger flower buds with an elongated conical shape, larger pollen and seed sizes, and distinct pollen and seed morphology. Furthermore, *R. pseudochrysanthum* ssp. morii var. *taitunense* exhibits a restricted and localized distribution, occurring exclusively in low-elevation mountainous areas of Northern Taiwan. Overall, our results indicate that this taxon has undergone clear morphological differentiation from other taxa within the *R. pseudochrysanthum* species complex.

## Introduction

*Rhododendron* subg. *Hymenanthes* (Blume) K.Koch belongs to the genus *Rhododendron* L. in the family Ericaceae Juss. They are also known as elepidote rhododendrons and they account for approximately one-quarter of the species diversity within the genus. This subgenus is classified into one section and 24 subsections (Chamberlain 1982), comprising around 270 species worldwide. It is primarily distributed in Asia, with additional occurrences in Europe and North America. China has 259 *Rhododendron* subg. *Hymenanthes* species (Fang et al. 2005). Most phylogenetic studies indicate that this subgenus forms a monophyletic group (Kron 1997; Kurashige et al. 1998, 2001; Milne 2004).

*Rhododendron rubropunctatum* Hayata was described by Hayata (1913) based on a specimen collected by S. Sasaki from Chihsingshan in Northern Taiwan. This taxon was later reported from Taiwan by several authors, including Kanehira (1917), Sasaki (1928), Masamune (1936), Kanehira (1936), and Masamune (1954). However, Wilson (1925), Li (1963), Hsu (1973), Ying (1976), and Li (1978) observed that both *R. rubropunctatum* Hayata and *R. hyperythrum* Hayata possess prominent glandular structures on the abaxial leaf surface, and consequently treated *R. rubropunctatum* Hayata as a synonym of *R. hyperythrum* Hayata.

In 1981, Yamazaki noted that a *Rhododendron* species occurring in the Tatun Volcanic Group of Northern Taiwan, including on the slopes of Chihsingshan and Tatunshan, was initially published as *R. rubropunctatum* by Hayata (1913). He pointed out that this name is a later homonym of *R. rubropunctatum* H. Lév. & Vaniot (1911), and thus, illegitimate. Yamazaki also noted that Wilson (1925) replaced the name with *R. hyperythrum* Hayata, which led to further taxonomic confusion (Yamazaki 1981). Nevertheless, due to the revolute leaf margins distinct from other taxa, Yamazaki renamed the taxon as *R. pseudochrysanthum* Hayata ssp. morii (Hayata) Yamazaki var. *taitunense* Yamazaki.

Later, Lu and Yang (1989) and Li et al. (1998) considered the reddish glands on the abaxial leaf surface to lack taxonomic significance and treated *R. rubropunctatum* Hayata, *R. morii* Hayata, and *R. pseudochrysanthum* Hayata ssp. morii (Hayata) Yamazaki var. *taitunense* Yamazaki as synonyms of *R. pseudochrysanthum* Hayata. In the same treatment, Li et al. (1998) recognized only three taxa in the *Rhododendron* subgenus *Hymenanthes* in Taiwan: *R. formosanum* Hemsl., *R. hyperythrum* Hayata, and *R. pseudochrysanthum* Hayata.

Furthermore, due to continuous morphological variation and the absence of clear genetic differentiation among *R. pseudochrysanthum* Hayata ssp. morii (Hayata) Yamazaki var. *taitunense* Yamazaki, *R. morii* Hayata, *R. pseudochrysanthum* Hayata, and *R. hyperythrum* Hayata, these four taxa have been collectively referred to as the *Rhododendron pseudochrysanthum* species complex (Lu and Yang 1989; Chung et al. 2007; Hsieh 2007; Liang 2011; Chen et al. 2014; Cao et al. 2022).

Pollen morphology is considered to exhibit a high degree of genetic conservatism, with intrinsic features such as shape, surface ornamentation, and the number of germination pores commonly used for plant classification and identification (Zhou et al. 2008). Moreover, it is informative for both taxonomic delimitation and phylogenetic analysis within certain groups of Ericaceae (Kron et al. 2002; Sarwar 2007). Within a single subgenus, pollen characters can be particularly valuable for the recognition of sections and subsections, for instance, in *Rhododendron* sect. *Ponticum* G. Don, although all taxa produce tetrad pollen grains, variations in pollen size and exine sculpture have been reported, reflecting meaningful interspecific differentiation (Huang 1972; Vasanthy and Pocock 1987; Fuhsiung et al. 1995; Mao et al. 2000; Terzioğlu et al. 2001; Gao et al. 2002; Wang et al. 2006; Zhang et al. 2009; Miyoshi et al. 2011). In *Rhododendron* species, seed morphology, size, and the presence or absence of appendages are closely related to plant habits, primarily reflecting the systematic differentiation among taxa within the genus (Geng 2014). Moreover, the width and shape of seed wings vary significantly across different groups (Ding et al. 1995; Wang et al. 2007).

Previous studies on the *Rhododendron pseudochrysanthum* species complex have focused primarily on external morphological descriptions. In this study, we re-examine morphological differentiation within the complex by incorporating a broader set of macromorphological characters, as well as pollen and seed morphological traits.

## Materials and methods

### Samples collection and morphological comparison

The materials used in this study were primarily collected from wild populations of the *R. pseudochrysanthum* species complex in Taiwan (Table 1). Fieldwork included photography, GPS point recording, and fixation of fresh leaves and flowers. External morphological traits, such as leaves, flowers, floral buds, and seeds, were observed and compared among the different taxa. Macroscopic features were documented using a digital camera equipped with a macro lens (Nikon D7500 + Nikon AF-S DX NIKKOR 40 mm f/2.8G Micro). At the same time, finer structures were examined under a high-magnification stereomicroscope (KEYENCE VHX-7020).

**Table 1.** Materials collected of the *R. pseudochrysanthum* species complex.

taxon	location	code	lat. (°N), long. (°E)	altitude (m)	Specimens examined
*R. pseudochrysanthum* ssp. morii var. *taitunense*	Mt. Qixing	taitun_QXS	25.17°N, 121.55°E	972	ZYR298
	Mt. Wuzhi	taitun_WZS	25.14°N, 121.62°E	635	ZYR257
	Mt. Bijia	taitun_BJS	24.97°N, 121.62°E	580	ZYR266
	Mt. Caigongkeng	taitun_CGK	25.19°N, 121.52°E	864	ZYR271
	Mt. Banping	taitun_BPS	25.10°N, 121.87°E	621	ZYR332
	Mt. Canguangliao	taitun_CGL	25.09°N, 121.87°E	717	ZYR270
	Mt. Xin	taitun_XS	25.13°N, 121.63°E	481	ZYR335
*R. morii*	Mt. Jiali	morii_JLS	24.51°N, 121.01°E	2,026	ZYR30
	Yushan Trail 5 km	morii_YS	23.47°N, 120.93°E	2,963	ZYR280
	Tataka	morii_TTK	23.48°N, 120.89°E	2,820	ZYR275
	Mt. Taman	morii_TMS	24.71°N, 121.45°E	2,109	ZYR222
	Mt. Xinan	morii_XNS	23.09°N, 120.81°E	2,439	ZYR345
	Beinanzhu	morii_BNS	23.07°N, 120.86°E	2,551	ZYR337
	Tefuya	morii_TFY	23.47°N, 120.82°E	2,580	ZYR375
	Mt. Xue 4 km	morii_XUE	24.39°N, 121.28°E	3,007	ZYR69
	Mt. Taiping	morii_TPS	24.49°N, 121.53°E	1,979	ZYR66
*R. pseudochrysanthum*	Hehuan East Peak	pseudo_HHE	24.14°N, 121.28°E	3,353	ZYR297
	Hehuan Road Side	pseudo_HHR	24.16°N, 121.28°E	3,084	ZYR350
	Mt. Xue	pseudo_XUE	24.39°N, 121.24°E	3,596	ZYR113
	Yushan Main Peak	pseudo_YS	23.47°N, 120.96°E	3,834	ZYR86
	Nanhu Main Peak	pseudo_NHM	24.36°N, 121.44°E	3,709	ZYR366
*R. hyperythrum*	Nanhu Cirque	hypery_NHC	24.37°N, 121.44°E	3,400	ZYR364
	Nanhu 18.6 km	hypery_NH18k	24.38°N, 121.44°E	3,460	ZYR367
	Nanhu 17 km	hypery_NH17k	24.38°N, 121.43°E	3,320	ZYR370
	Shenmazheng	hypery_SMZ	24.38°N, 121.44°E	3,457	ZYR362
	Wuyan Peak	hypery_WYF	24.38°N, 121.44°E	3,544	ZYR369

### Herbarium resources

Herbarium acronyms from Index Herbariorum were used in this study (Thiers 2026, continuously updated). Voucher specimens collected for the current study were deposited in the herbarium of the Department of Forestry, National Chung-Hsing University, Taiwan (**TCF**). Physical and digital specimens in several herbaria were also examined; physical specimens: Taiwan Forestry Research Institute (**TAIF**), Herbarium of Taiwan Biodiversity Research Institute, Taiwan (**TAIE**), National Taiwan University, Taiwan (**TAI**), National Chung-Hsing University, Taiwan (**TCF**); and digital specimens: Department of Forestry and Natural Resources, National Chia-Yi University, Taiwan (**CHIA**), Provincial Pingtung Institute, Taiwan (**PPI**), the Royal Botanic Gardens, Kew (**K**), Herbarium of Academia Sinica, Taiwan (**HAST**), and Herbarium of the University of Tokyo, Japan (**TI**).

### Pollen morphology

Fresh stamens were collected and fixed in 70% ethanol. During the collection, pollen was extracted from the anthers and placed into a 1.5 ml centrifuge tube containing 70% ethanol. After a single centrifugation step, the supernatant was removed, and a mixture of 30 ml 2,2-dimethoxypropane (DMP) and 1 drop of 1M HCl was added. The sample was incubated for approximately 2 hours, followed by an acetone substitution for at least 1 hour. Subsequently, the samples underwent critical point drying (CPD) (Halbritter 1998). The mounted samples were then gold-coated for 100 seconds at 15 mA using a Quorum SC7620 sputter coater (Quorum Technologies, Laughton, UK). Finally, observations and imaging were performed using a scanning electron microscope (Hitachi S-3400N) at 15 kV.

The morphological characteristics of pollen were described with reference to Erdtman (1952), Sarwar (2007), and Sarwar and Takahashi (2013). Observations included the shape and size of tetrad pollen, as well as the following measurements (in µm): Tetrad diameter (D), Polar length (P), Equatorial diameter (d for tetrads; E for monads), Colpus length (2f for tetrads; L for monads), P/d ratio (polar to equatorial diameter ratio), D/d ratio (tetrad diameter to equatorial diameter ratio), 2f/D ratio (colpus length to tetrad diameter ratio), and Apocolpial exine sculpture.

### Seed morphology

Mature dehiscent capsules of each taxon were collected from various sampling sites. A total of 3–5 capsules were placed in an oven at 50 °C for at least 48 hours. Thirty mature seeds were randomly sampled for each taxon and observed under a high-magnification dissecting microscope (KEYENCE VHX-7020) to examine and document seed size and appendages. The morphological characteristics of the seeds were described with reference to Barthlott (1981, 1990) and Geng (2014), and the following measurements (in mm) were noted: seed length (L) and seed width (W).

### Distribution map

The collection site information of this study, including GPS coordinates and elevation, was integrated. The distribution map was adapted from Lin (2018), and the distribution points of each taxon were plotted using QGIS ver. 3.4. Additionally, the study referred to the geographical and climatic regions of Taiwan as defined by Su (1985) and the montane vegetation zones to analyze the vertical and horizontal distribution patterns of each taxon.

### Data analysis

Quantitative traits were measured using ImageJ software. Statistical analyses were conducted in R 4.3.2 using the stats package (R Core Team 2024). First, normality was assessed using the Shapiro-Wilk test (shapiro.test) to determine whether the quantitative trait values followed a normal distribution across taxa (*p* < 0.05 indicates non-normal distribution). If traits followed a normal distribution, one-way ANOVA was performed using the aov function, followed by a Tukey HSD post-hoc test (TukeyHSD, *p* ≤ 0.05) to assess significant differences among taxa. If traits did not follow a normal distribution, nonparametric analysis was conducted using the Kruskal-Wallis test (kruskal.test) to determine differences among taxa, followed by a pairwise Wilcoxon rank-sum test (pairwise.wilcox.test, *p* ≤ 0.05) for post-hoc comparisons. Boxplots were generated using the ggboxplot function from the ggplot2 package (Wickham 2016) to visualize data distribution.

To investigate the clustering results of taxa based on characteristic variables such as pollen and seeds, cluster analysis was conducted using R 4.3.2. The stats package (R Core Team 2024) was used with the dist function to construct a distance matrix based on Euclidean distance to determine the proximity between data points. Hierarchical cluster analysis was then performed using the hclust function with Ward’s minimum variance method. Finally, a dendrogram was plotted using the plot function from the graphics package (R Core Team 2024).

## Results and discussion

### Macromorphological differences

**Figure 1.**
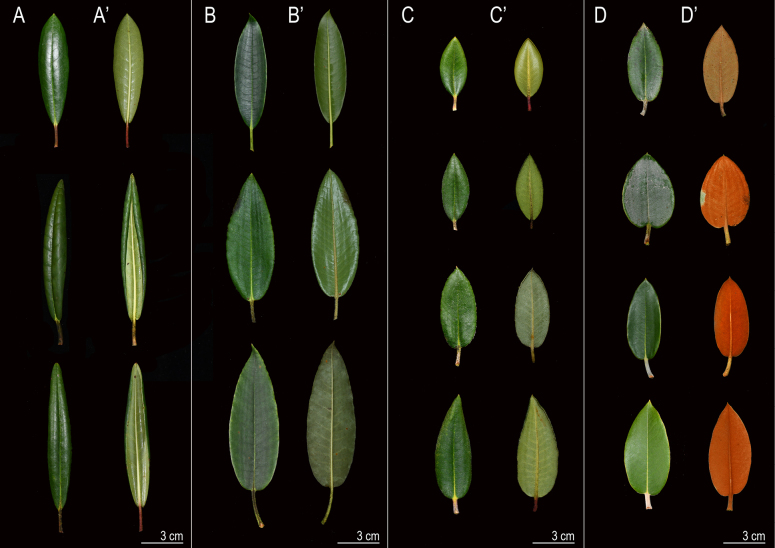
Leaf morphology of *Rhododendron pseudochrysanthum* species complex. **A**. *R. pseudochrysanthum* Hayata ssp. morii (Hayata) Yamazaki var. *taitunense* Yamazaki; **B**. *R. morii* Hayata; **C**. *R. pseudochrysanthum* Hayata; **D**. *R. hyperythrum* Hayata.

The leaf morphology of the *R. pseudochrysanthum* species complex is highly variable (Fig. 1, Table 2), exhibiting shapes such as narrow elliptic, lanceolate, oblanceolate, and narrow ovate. Leaf base morphology is also diverse, and many traits show considerable intraspecific variation. Although leaf length differs significantly among some taxa (Fig. 4, Table 2), leaf size and shape can be influenced by habitat conditions or climate, and even within a single individual, variation may occur due to light exposure (Geng 2014). Therefore, leaf size, shape, and base morphology alone cannot be used as reliable taxonomic criteria. In terms of epidermal appendages, glands were present across all four taxa, consistent with findings by Lu and Yang (1989), indicating that this trait is not a valid diagnostic character. However, trichomes exhibited high diversity among the four taxa, suggesting that this macromorphological feature may serve as a useful characteristic for taxonomic identification.

**Table 2.** Macromorphological differences within the *Rhododendron pseudochrysanthum* species complex.

taxon	*R. pseudochrysanthum* ssp. morii var. *taitunense*	*R. morii*	*R. pseudochrysanthum*	*R. hyperythrum*
traits				
Leaf length	9.482 ± 1.203^a^	9.264 ± 1.641^a^	5.272 ± 0.761^b^	5.370 ± 0.988^b^
Leaf width	2.465 ± 0.589^ab^	2.713 ± 0.484^b^	2.080 ± 0.408^c^	2.371 ± 0.393^a^
Leaf length/width	4.141 ± 1.186^a^	3.454 ± 0.367^b^	2.634 ± 0.307^c^	2.287 ± 0.383^b^
Leaf lateral vein pairs	10–15	12–19	12–15	11–14
Leaf shape	narrowly elliptic- oblanceolate	narrowly elliptic- lanceolate	narrowly ovate	narrowly ovate
Leaf margin	strongly revolute	flat to revolute	revolute	revolute
Leaf base	obtuse-acute	obtuse-acute	obtuse-acute	obtuse-cordate
Leaf trichome	hairless	tufted hair on abaxial midrib	glandular hair on abaxial midrib	branched hair on leaf abaxial
Flower bud length	3.615 ± 0.372^a^	2.170 ± 0.288^b^	2.019 ± 0.141^b^	1.928 ± 0.178^b^
Flower bud width	1.554 ± 0.240^a^	1.260 ± 0.083^b^	1.165 ± 0.096^c^	1.215 ± 0.069^b^
Flower bud length/width	2.367 ± 0.331^a^	1.722 ± 0.190^bc^	1.742 ± 0.162^b^	1.592 ± 0.173^c^
Style hair type	glandular hairs	glandular or puberulent	glandular or puberulent	glandular or puberulent
Ovary hair type	glandular hairs	glandular or puberulent	glandular or puberulent	glandular or puberulent
calyx	lobes 5, triangular, with glandular hairs	lobes 5, triangular, with glandular hairs	lobes 5, triangular, with glandular hairs	lobes 5, triangular, with glandular hairs
Pedicel hair type	glandular hairs	glandular or puberulent	glandular or puberulent	glandular or puberulent
Locule number	6–10	6–8	6–7	6–7
Stamen number	10–12	10–12	10–12	10–12

Mean ± SE (n = 5); Unit = centimeter (cm). Different letters indicate statistically significant differences (*p* < 0.05).

**Figure 2.**
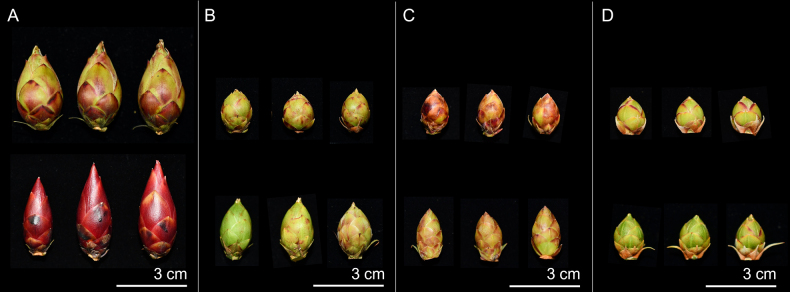
Flower bud morphology of *Rhododendron pseudochrysanthum* species complex. **A**. *R. pseudochrysanthum* Hayata ssp. morii (Hayata) Yamazaki var. *taitunense* Yamazaki; **B**. *R. morii* Hayata; **C**. *R. pseudochrysanthum* Hayata; **D**. *R. hyperythrum* Hayata.

**Figure 3.**
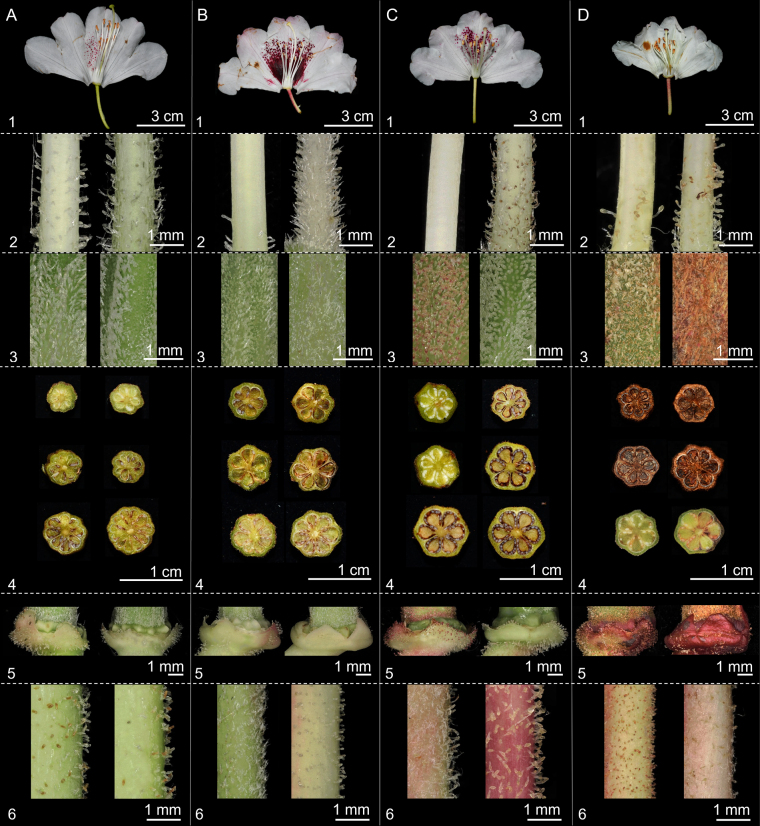
Flower morphology of *Rhododendron pseudochrysanthum* species complex. **A**. *R. pseudochrysanthum* Hayata ssp. morii (Hayata) Yamazaki var. *taitunense* Yamazaki; **B**. *R. morii* Hayata; **C**. *R. pseudochrysanthum* Hayata; **D**. *R. hyperythrum* Hayata: 1, flower; 2, style; 3, ovary; 4, ovary locules; 5, calyx; 6, pedicel.

**Figure 4.**
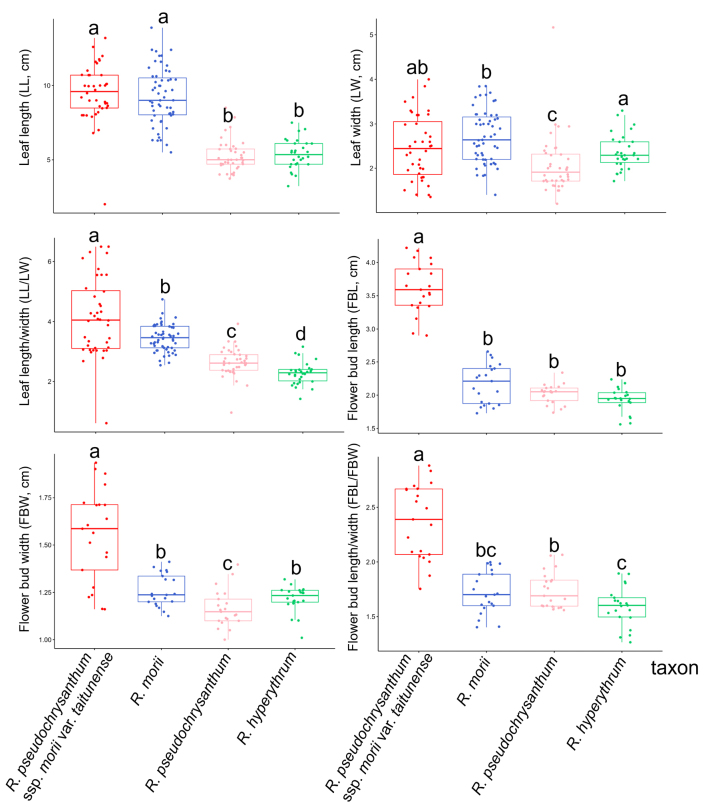
Boxplot comparing the leaf and flower bud characteristics of *Rhododendron pseudochrysanthum* species complex. Different letters in each boxplot indicate statistically significant differences (*p* < 0.05).

Within the *R. pseudochrysanthum* species complex, traits such as the indumentum on the style, ovary, calyx, pedicel, and stamens, as well as the number of ovary locules, exhibit variation among individuals within each taxon. Floral color also varies depending on environmental conditions. However, *R. pseudochrysanthum* ssp. morii var. *taitunense* consistently possesses a higher number of ovary locules compared to the other taxa. (Fig. 3, Table 2). Therefore, some of these characters are not suitable as diagnostic features for species delimitation within the species complex. However, in terms of floral bud size, *R. pseudochrysanthum* ssp. morii var. *taitunense* displays significantly greater bud length, width, and length-to-width ratio (*p* < 0.05) compared to the other taxa (Figs 2, 4). In contrast, *R. morii*, *R. pseudochrysanthum*, and *R. hyperythrum* show no significant differences in floral bud dimensions (Fig. 4, Table 2).

### Pollen morphology

**Figure 5.**
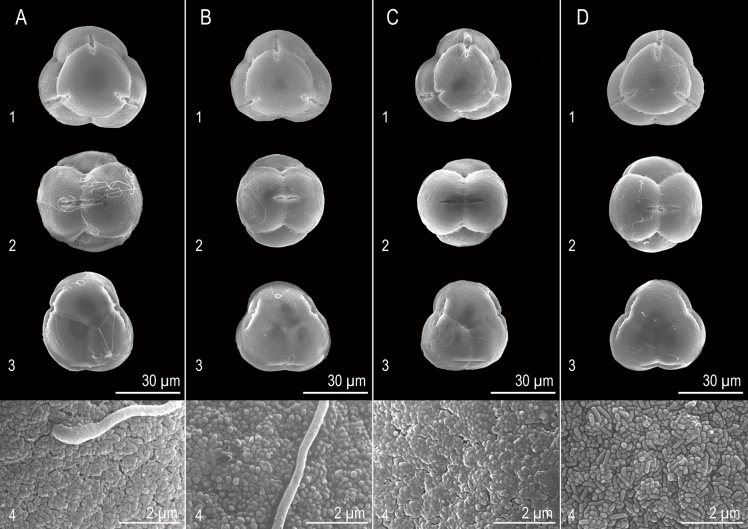
Pollen morphology of the *Rhododendron pseudochrysanthum* species complex. **A**. *R. pseudochrysanthum* Hayata ssp. morii (Hayata) Yamazaki var. *taitunense* Yamazaki; **B**. *R. morii* Hayata; **C**. *R. pseudochrysanthum* Hayata; **D**. *R. hyperythrum* Hayata: 1, tetrads polar plane; 2, tetrads equatorial plane; 3, tetrads polar plane; 4, apocolpial exine sculpture.

The pollen grains of the *R. pseudochrysanthum* species complex are all tetrads, arranged in a tetrahedral configuration, and exhibit heteropolarity (Fig. 5). The average diameter of the tetrads ranges from 40.243 ± 0.848 µm to 45.090 ± 1.096 µm; the average polar axis length ranges from 19.525 ± 0.465 µm to 21.804 ± 0.638 µm; the average equatorial diameter ranges from 29.469 ± 1.464 µm to 32.462 ± 1.622 µm; and the average colpus length ranges from 14.035 ± 1.362 µm to 19.723 ± 0.764 µm (Table 3). The P/d ratio ranges from 0.666 ± 0.038 to 0.687 ± 0.020; the D/d ratio ranges from 1.363 ± 0.042 to 1.393 ± 0.062; and the 2f/D ratio ranges from 0.350 ± 0.037 to 0.439 ± 0.019 (Table 3). According to Erdtman (1952), based on the classification of pollen grain size and shape, these pollen grains are categorized as medium-sized (Mediae, 25–50 µm) and oblate in shape. They are 3-colporate, with a circular outline in polar view.

**Table 3.** Pollen morphological differences of the *R. pseudochrysanthum* species complex.

taxon	*R. pseudochrysanthum* ssp. morii var. *taitunense*	*R. morii*	*R. pseudochrysanthum*	*R. hyperythrum*
traits				
Tetrad diameter (D)	45.090 ± 1.096^a^	40.988 ± 0.896^b^	40.369 ± 0.882^bd^	40.243 ± 0.848^d^
Polar length (P)	21.804 ± 0.638^a^	20.050 ± 0.569^b^	19.525 ± 0.465^c^	20.325 ± 0.403^b^
Equatorial diameter (d)	32.462 ± 1.622^a^	29.894 ± 1.139^b^	29.469 ± 1.464^b^	29.621 ± 0.675^b^
Colpus length (2f)	19.723 ± 0.764^a^	16.122 ± 0.746^b^	15.991 ± 1.332^b^	14.035 ± 1.362^c^
P/d ratio	0.675 ± 0.048^ab^	0.673 ± 0.034^ab^	0.666 ± 0.038^a^	0.687 ± 0.020^b^
D/d ratio	1.393 ± 0.062^a^	1.374 ± 0.045^a^	1.376 ± 0.050^a^	1.363 ± 0.042^a^
2f/D ratio	0.439 ± 0.019^a^	0.394 ± 0.016^b^	0.397 ± 0.032^b^	0.350 ± 0.037^c^
apocolpial exine sculpture	gemmate	gemmate	gemmate	gemmate

Mean ± SE (n = 10); Unit = micrometer (μm). Different letters indicate statistically significant differences (*p* < 0.05).

**Figure 6.**
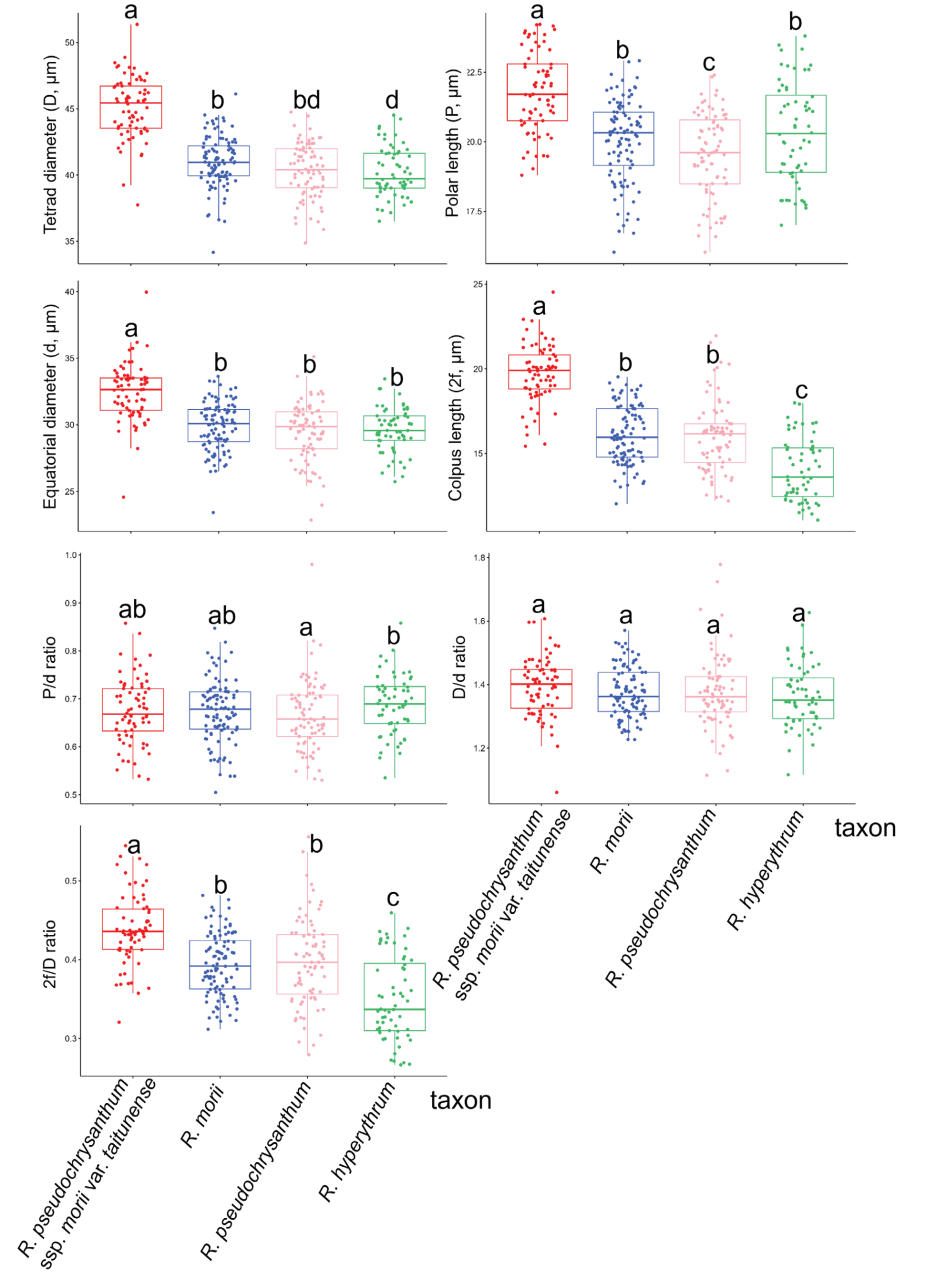
Boxplot comparing the pollen grain characteristics of the *R. pseudochrysanthum* species complex. Different letters in each boxplot indicate statistically significant differences (*p* < 0.05).

Overall, *R. pseudochrysanthum* ssp. morii var. *taitunense* exhibits significant differences (*p* < 0.05) in all quantitative pollen traits (except for the P/d and D/d ratios) compared to the other three taxa within the *R. pseudochrysanthum* species complex (Fig. 6). According to the study by Sarwar and Takahashi (2013), the apocolpial exine sculpture of pollen in the *R. pseudochrysanthum* species complex is consistently of the gemmate type (Fig. 5).

### Seed morphology

**Figure 7.**
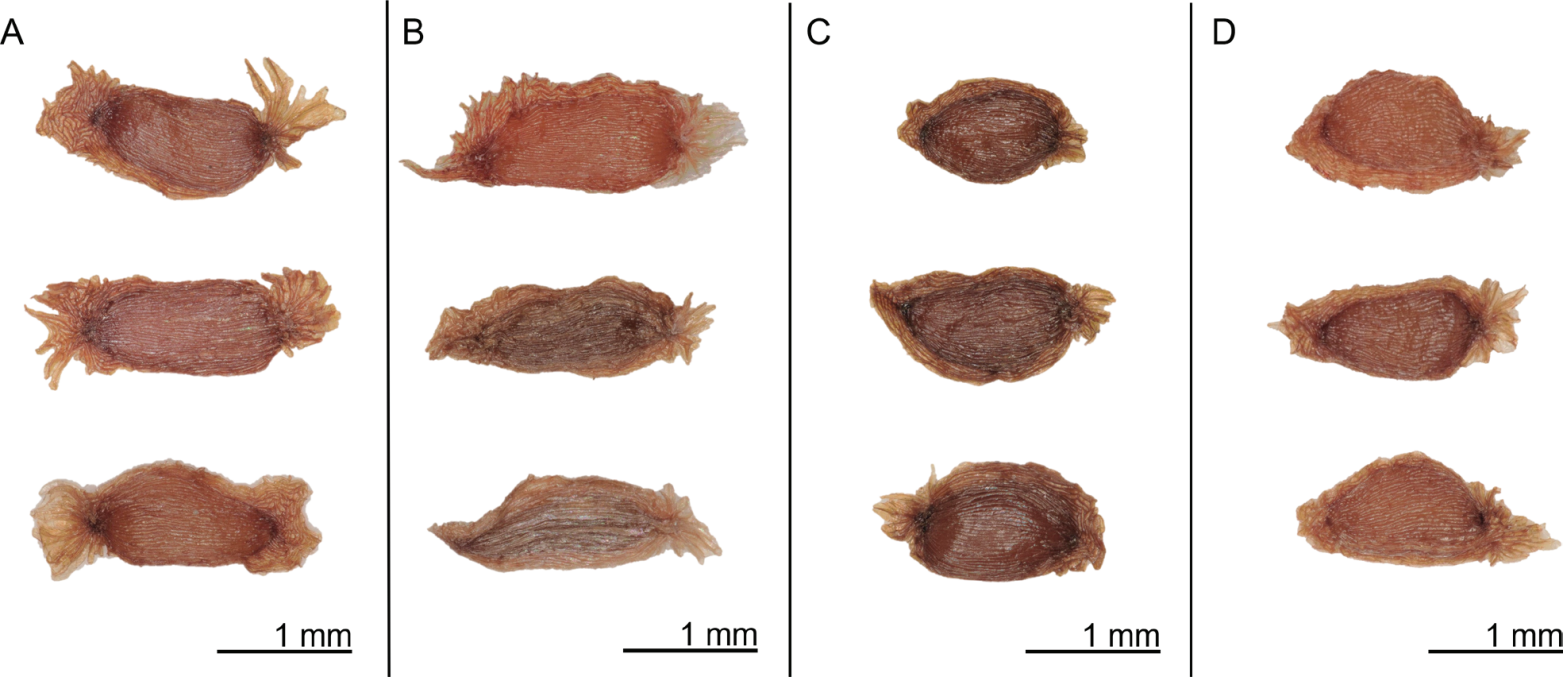
Seed morphology of the *Rhododendron pseudochrysanthum* species complex. **A**. *R. pseudochrysanthum* Hayata ssp. morii (Hayata) Yamazaki var. *taitunense* Yamazaki; **B**. *R. morii* Hayata; **C**. *R. pseudochrysanthum* Hayata; **D**. *R. hyperythrum* Hayata.

**Figure 8.**
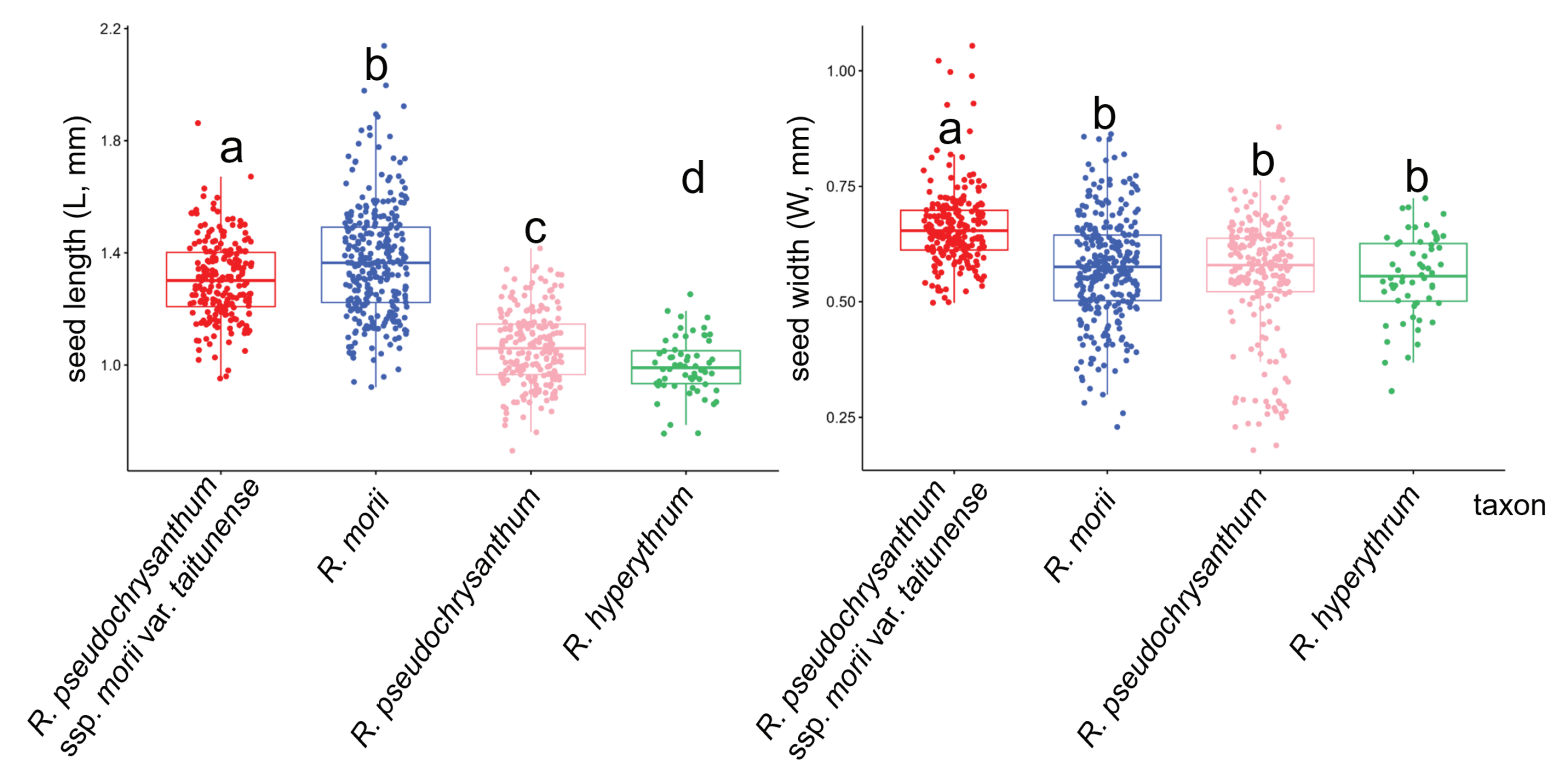
Boxplot comparing the seed characteristics of the *R. pseudochrysanthum* species complex. Different letters in each boxplot indicate statistically significant differences (*p* < 0.05).

The fruit of the *R. pseudochrysanthum* species complex is a septicidal capsule, and the seeds are elongate or narrowly elliptic in shape (Fig. 7). The average seed length ranges from 1.062 ± 0.095 mm to 1.371 ± 0.146 mm, and the average seed width ranges from 0.552 ± 0.121 mm to 0.662 ± 0.058 mm (Table 4). Within the *R. pseudochrysanthum* species complex, *R. pseudochrysanthum* ssp. morii var. *taitunense* shows significant differences (*p* < 0.05) in both seed length and width when compared to *R. morii*, *R. pseudochrysanthum*, and *R. hyperythrum* (Fig. 8). However, no significant differences in seed width were observed among *R. morii*, *R. pseudochrysanthum*, and *R. hyperythrum*.

**Table 4.** Seed morphological differences of the *R. pseudochrysanthum* species complex.

taxon	*R. pseudochrysanthum* ssp. morii var. *taitunense*	*R. morii*	*R. pseudochrysanthum*	*R. hyperythrum*
traits				
Seed length	1.303 ± 0.070^a^	1.371 ± 0.146^b^	1.062 ± 0.095^c^	0.996 ± 0.102^d^
Seed width	0.662 ± 0.058^a^	0.574 ± 0.092^b^	0.552 ± 0.121^b^	0.552 ± 0.094^b^
Seed shape	narrowly elliptic	narrowly elliptic	elongate	elongate

Mean ± SE (n = 30); Unit = millimeter (mm). Different letters indicate statistically significant differences (*p* < 0.05).

As elevation increases, wind becomes stronger, facilitating more effective seed dispersal. Therefore, *Rhododendron* species at higher elevations often possess narrower seeds and seed wings. Since stronger winds at high altitudes can effectively disperse seeds, there is less selective pressure to develop elaborate dispersal structures (Howe and Smallwood 1982). Nonetheless, *R. pseudochrysanthum* and *R. hyperythrum*, both distributed at high elevations, still exhibit prominent wing-like seed appendages (Fig. 7).

### Cluster analysis

**Figure 9.**
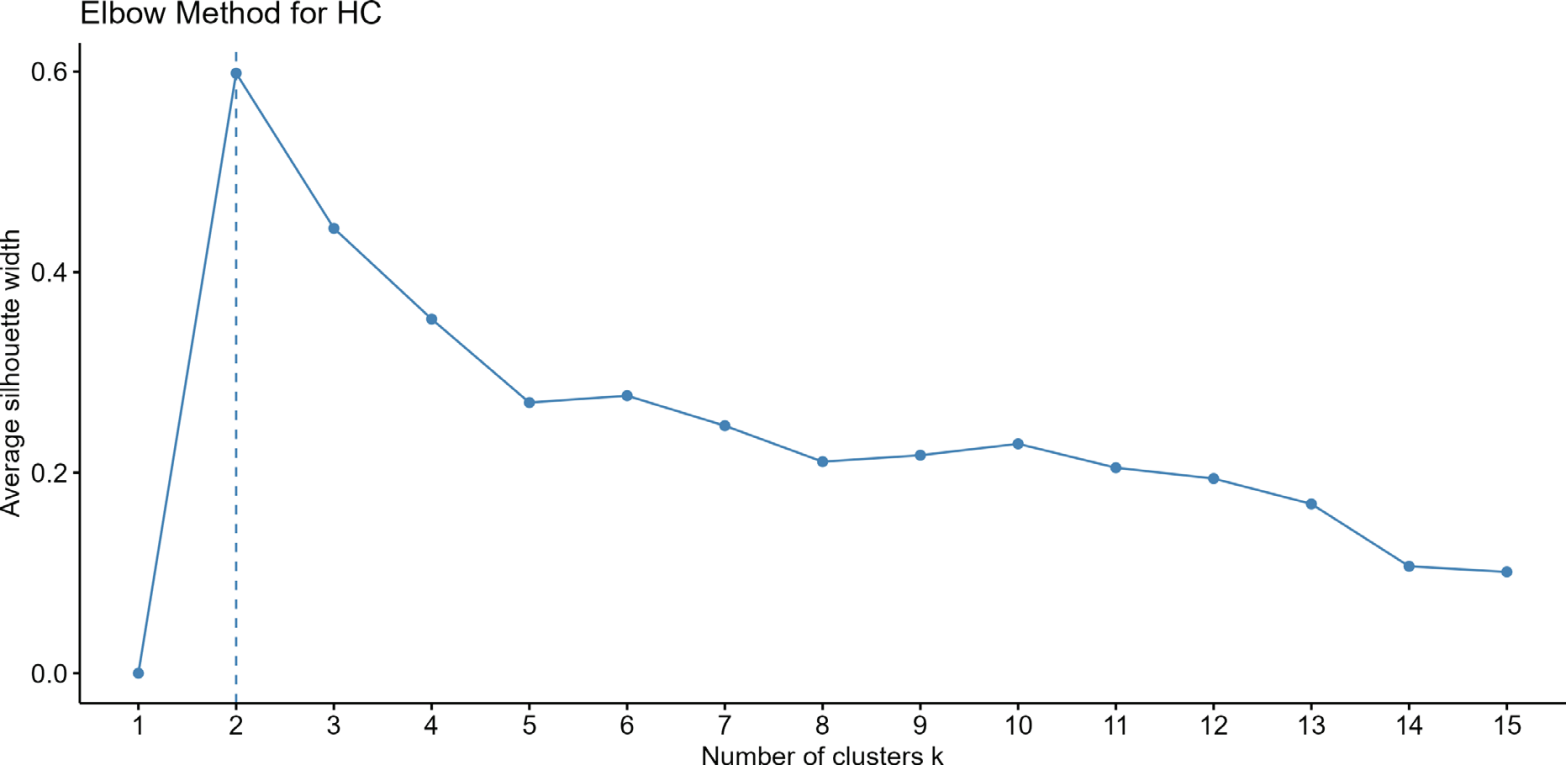
Elbow Method for Determining Optimal Number of Clusters in Hierarchical Clustering. Number of clusters k = 2.

**Figure 10.**
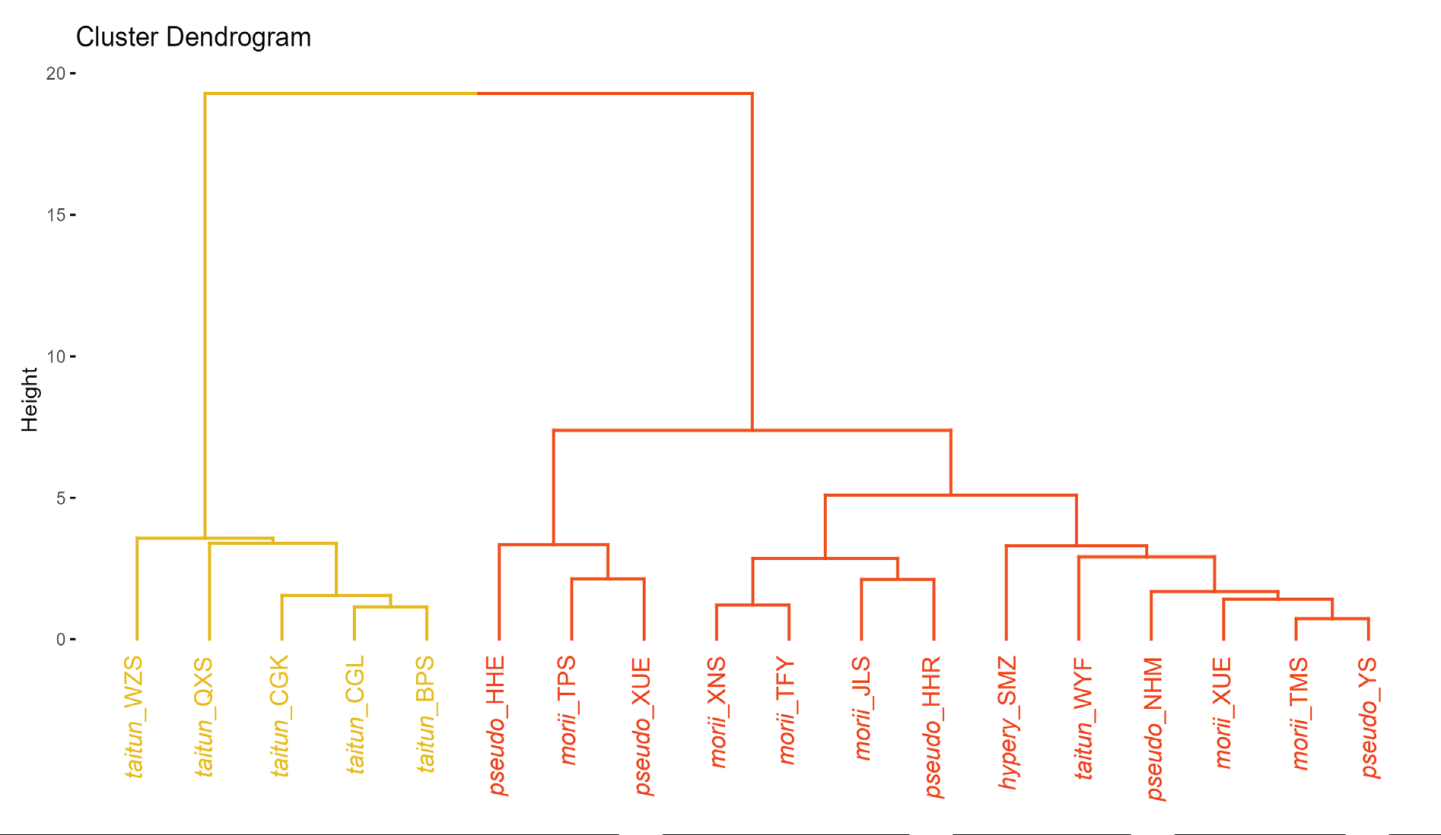
Cluster analysis of reproductive traits. The yellow lines are *R. pseudochrysanthum* ssp. morii var. *taitunense*, and the orange lines are *R. morii*, *R. pseudochrysanthum*, and *R. hyperythrum*.

To determine the optimal number of clusters, we employed the Elbow Method in conjunction with average silhouette width as an evaluation metric, using hierarchical clustering (HC) analysis. The results indicated that when the number of clusters was set to two, the average silhouette width reached its maximum value (approximately 0.8; Fig. 9), suggesting the highest intra-cluster similarity and inter-cluster dissimilarity at this point. Therefore, based on this analysis, dividing the samples into two clusters was considered the optimal solution, further supporting a clear distinction between *R. pseudochrysanthum* ssp. morii var. *taitunense* and the other taxa in terms of pollen and seed characteristics (Fig. 10). This result indicates that *R. pseudochrysanthum* ssp. morii var. *taitunense* is distinct from the other three taxa within the *R. pseudochrysanthum* species complex in terms of reproductive traits.

### Distribution and habitat

**Figure 11.**
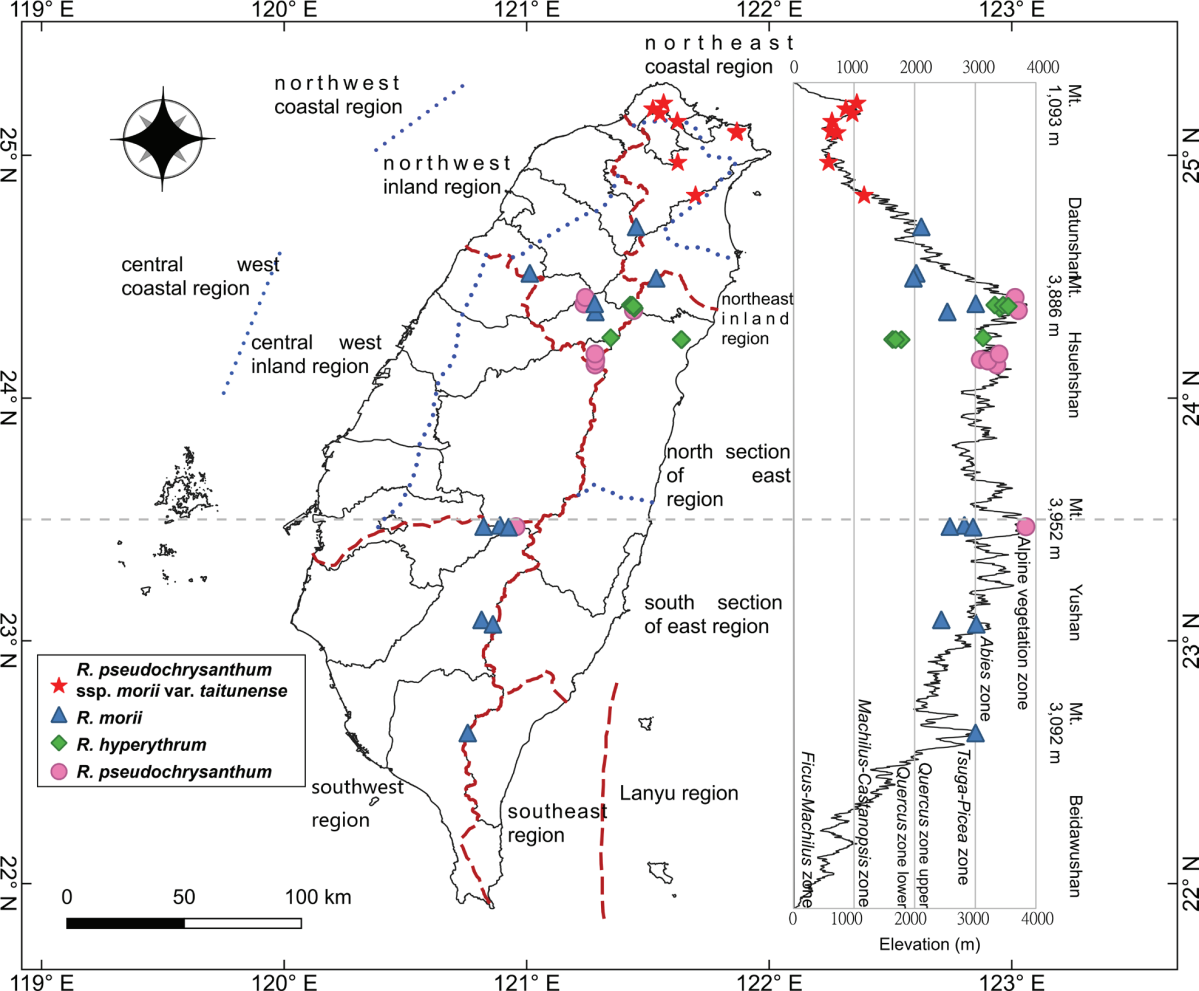
Distribution map of *Rhododendron pseudochrysanthum* species complex in Taiwan.

Within the *Rhododendron pseudochrysanthum* species complex, *R. pseudochrysanthum* ssp. morii var. *taitunense* shows a distinct geographic distribution compared with other taxa, occurring in the Datun Volcanic Group, Keelung Volcanic Group, Shiding District, and Wulai District at elevations of 440–1,200 m (Fig. 11). Currently, it is known from Mt. Qixing and Mt. Wuzhi in Taipei City, as well as Mt. Zhuzi, Mt. Caigongkeng, Mt. Xin, Mt. Canguangliao, Mt. Banping, Mt. Bijia, and Mt. Hongludi in New Taipei City. This taxon primarily grows along ridges, cliff edges, or in open forest habitats, whereas the other three taxa are mainly distributed in mid- to high-elevation mountainous regions of Taiwan.

## Conclusions

In summary, based on the literature review, the geographical distribution, and morphological analyses, we found that *R. pseudochrysanthum* ssp. morii var. *taitunense* is clearly distinguishable from other taxa within the *R. pseudochrysanthum* species complex by its strongly revolute mature leaves, larger flower buds with a long conical shape (over 3 cm), and larger pollen grains. Furthermore, the restricted and localized distribution of this taxon solely to the low-elevation mountainous areas of Northern Taiwan further supports its distinctness.

In addition, we found that *R. morii*, *R. pseudochrysanthum*, and *R. hyperythrum* are difficult to delineate taxonomically based on certain morphological characteristics, such as floral morphology, pollen, and seed. This complexity is compounded by the occurrence of intermediate individuals between *R. pseudochrysanthum* and *R. hyperythrum* in high-elevation areas, making species delineation challenging. Therefore, more in-depth studies on these remaining taxa are planned for the future, including expanding sampling efforts and incorporating molecular evidence.

## Additional information

### Use of AI

AI was used for academic English editing.

### Author ORCIDs

Ya-Rong Zheng https://orcid.org/0009-0007-7991-5260

Yen-Hsueh Tseng https://orcid.org/0000-0003-3644-1863

Hsy-Yu Tzeng https://orcid.org/0000-0003-0952-6255

### Data availability

All of the data that support the findings of this study are available in the main text.
